# NMR-based fecal metabolomics fingerprinting as predictors of earlier diagnosis in patients with colorectal cancer

**DOI:** 10.18632/oncotarget.8762

**Published:** 2016-04-16

**Authors:** Yan Lin, Changchun Ma, Chengkang Liu, Zhening Wang, Jurong Yang, Xinmu Liu, Zhiwei Shen, Renhua Wu

**Affiliations:** ^1^ Radiology Department, Second Affiliated Hospital, Shantou University Medical College, Shantou 515041, Guangdong, China; ^2^ Radiation Oncology, Affiliated Tumor Hospital, Shantou University Medical College, Shantou 515041, Guangdong, China; ^3^ Shantou University, Central Laboratory and NMR Unit, Shantou 515041, Guangdong, China; ^4^ Surgery Deparment, Second Affiliated Hospital, Shantou University Medical College, Shantou 515041, Guangdong, China

**Keywords:** colorectal cancer, ^1^H NMR spectroscopy, metabolomics, fecal profile, OPLS-DA

## Abstract

Colorectal cancer (CRC) is a growing cause of mortality in developing countries, warranting investigation into its earlier detection for optimal disease management. A metabolomics based approach provides potential for noninvasive identification of biomarkers of colorectal carcinogenesis, as well as dissection of molecular pathways of pathophysiological conditions. Here, proton nuclear magnetic resonance spectroscopy (^1^HNMR) -based metabolomic approach was used to profile fecal metabolites of 68 CRC patients (stage I/II=20; stage III=25 and stage IV=23) and 32 healthy controls (HC). Pattern recognition through principal component analysis (PCA) and orthogonal partial least squares-discriminant analysis (OPLS-DA) was applied on ^1^H-NMR processed data for dimension reduction. OPLS-DA revealed that each stage of CRC could be clearly distinguished from HC based on their metabolomic profiles. Successive analyses identified distinct disturbances to fecal metabolites of CRC patients at various stages, compared with those in cancer free controls, including reduced levels of acetate, butyrate, propionate, glucose, glutamine, and elevated quantities of succinate, proline, alanine, dimethylglycine, valine, glutamate, leucine, isoleucine and lactate. These altered fecal metabolites potentially involved in the disruption of normal bacterial ecology, malabsorption of nutrients, increased glycolysis and glutaminolysis. Our findings revealed that the fecal metabolic profiles of healthy controls can be distinguished from CRC patients, even in the early stage (stage I/II), highlighting the potential utility of NMR-based fecal metabolomics fingerprinting as predictors of earlier diagnosis in CRC patients.

## INTRODUCTION

Colorectal cancer (CRC) is one of the most prevalent types of cancer, ranking as the 3^rd^ most common malignancy and the 4^th^ leading cause of cancer death worldwide [1]. Patients with early stage CRC have significantly higher 5-year survival rates compared to patients diagnosed at later stages [2]. There is a need for better non-invasive clinical tools to improve detection of the disease in its early stages. Currently, preventive screening and detection methods for CRC rely upon clinical, endoscopic, histologic, and radiographic techniques that can be time-consuming, invasive and costly. Although colonoscopy remains the gold standard to diagnose CRC, it is invasive, expensive, and uncomfortable [3]. CT colonography is still improving its technical performance, which is however counterbalanced by radiation hazard and high cost. While non-invasive stool-based tests, such as fecal occult blood test (FOBT) and fecal immunochemical test (FIT), are convenient methods for screening CRC, their sensitivity are low, which reduce their reliability [4]. Fecal DNA (sDNA) [5] and microRNA [6] testings based on genetic alterations have been an area of active investigation since 1992, but they are costly and the sensitivity is low, making their reliability questionable. Therefore, it is essential to obtain an accurate, noninvasive, inexpensive and early diagnosis of CRC, for optimal disease management.

Metabolomics is an emerging field of research downstream of transcriptomics, genomics, and proteomics, concerned with the investigations of the biochemical processes that involve metabolites. As metabolites are present in readily-available biofluids, metabolomics has been applied to the diagnosis of many cancers, such as bladder [7], lung [8–10] and prostate [11]. Proton nuclear magnetic resonance (^1^H NMR) spectroscopy is a well-established, robust, reproducible, and cheap tool for quantifying metabolic profiles [12–14], which offers several advantages over other analytical techniques, including nondestructive analysis of samples, minimal sample preparation, and the ability to detect multiple metabolites within a single experiment [15, 16]. Biomarker assays characterized by NMR spectroscopy-based metabolomics for CRC detection have been developed for serum [17, 18] or urine [19], but these approaches may be limited because these fluids are anatomically remote from the gut mucosa in which CRC arises. There is evidence to suggest that microbial metabolism of proteins and amino acids by gut microbiota generate a variety of compounds, including branched chain fatty acids, indole and vitamin K [20], many of which elicit toxic effects on the lumen and contribute to CRC carcinogenesis through several immunologic and metabolic pathways [21–24]. Therefore, ^1^H-NMR spectroscopy-based metabolomic of human feces may be effective to investigate the microbiome and metabolic interactions to unravel CRC-associated metabolic alterations, since feces is anatomically attached to the colorectal epithelium and carries a large number of useful endogenous metabolites derived from gut microbial-host co-metabolism. In addition, large amount of exfoliated cells (approx 1.5 million per gram) from the colonic mucosa and colorectal tumor can be shed into the feces [25], which may provide a rich source for detecting tissue-specific metabolic biomarkers of CRC at the downstream fecal level.

Fecal metabolomics-based diagnosis by ^1^H NMR spectroscopy has shown the potential of this methodology in the assessment of systemic metabolic disturbances underlying CRC, as well as assisting with the diagnosis of disease [26–28]. Notably, short chain fatty acids (SCFAs) and some amino acids were identified as predominant fecal biomarkers for differentiating CRC patients from healthy individuals [26, 27]. However, there are questions that have yet to be fully elucidated: What sensitivity and specificity of the NMR-based fecal metabolomics is required to distinguish CRC patients from the healthy population? Do the changes in SCFAs and other fecal metabolites characteristically occur in the earlier stage of CRC? Do differences in fecal profiles occur across different populations due to differences in dietary, environmental and genetic factors? Answering these questions may glean some valuable understanding as to which fecal metabolites are associated with CRC, and therefore enhance the early screening of CRC patients.

In this study, fecal metabolomic profiles from Chinese CRC patients at various stages and healthy controls were obtained using ^1^H-NMR spectroscopy coupled with pattern recognition. Internal and external validations were performed to confirm the exact metabolic alterations that occur with each disease state. Our study observed distinct fecal metabolic signatures which were capable of discriminating early stage (stage I/II) CRC patients from healthy controls, leading to propose that NMR-based fecal metabolomics fingerprinting could be used as potential predictors of earlier diagnosis in patients with colorectal cancer.

## RESULTS

### Fecal metabolic profiles

**Figure 1:**
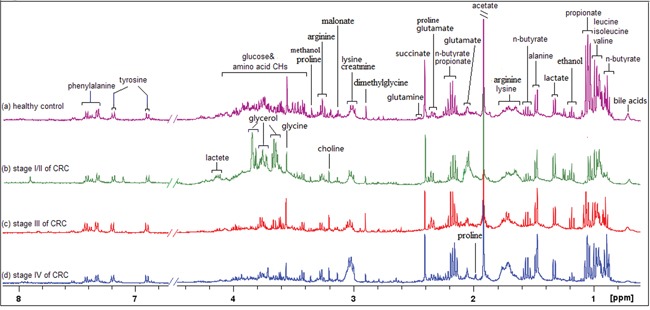
400 MHz representative 1H NMR spectra of fecal extracts obtained from healthy control **A.** stage I/II of CRC **B.** stage III of CRC(c) and stage IV of CRC, referenced to TSP (0.0 ppm).

Representative 1D ^1^H NMR spectra of fecal extracts obtained from healthy controls and different stages of CRC are shown in Figure 1. The standard one-dimension spectrum gave an overview of all metabolites. The major metabolites in the spectra were identified according to previous literatures [29–32] and Human Metabolome Database [33]. In all spectra, the aliphatic region at 0.6-4.5 ppm included prominent signals from water-soluble metabolites, such as amino acids (e.g., leucine, isoleucine, valine, alanine, lysine, dimethylglycine, asparate, tyrosine, glutamate, proline, succinate), SCFAs (e.g., acetate, propionate, butyrate), creatinine, ethanol, choline, lactate and glucose components.

### Pattern recognition analysis of CRC group and healthy controls

**Figure 2:**
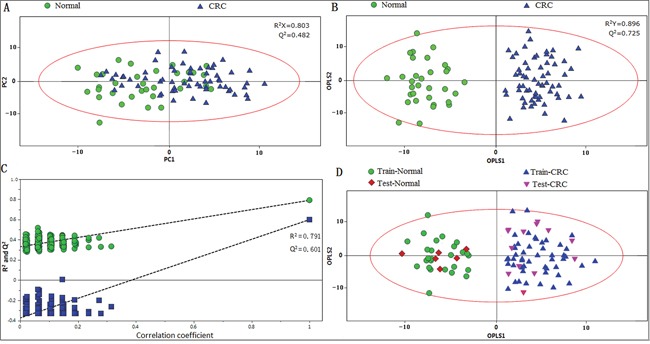
PR of fecal metabolomic profiles analyzed by 1H-NMR Spectrosocpy **A.** PCA scatter plot of fecal extract obtained from healthy controls (green dots) and CRC patients (blue triangles). **B.** OPLS-DA scatter plot based on the same samples. **C.** statistical validation of the corresponding OPLS-DA model by permutation analysis (200 times). **D.** scores plot of OPLS-DA prediction model. 80% of samples were applied to construct the model, and then used it to predict the remaining 20% of samples (“testing set”, healthy controls = 6; CRC patients=14). Red diamonds represent healthy controls and purple inverted triangles represent CRC.

Principal component analysis (PCA) was initially carried out to generate an overview of the variations between CRC patients and healthy controls, and some trends in differences were detected on the scores plot of first two principal components (PC) (Figure 2A). The majority of samples were located within the 95% confidence interval. To optimize the separation between the cancers and controls, orthogonal partial least squares-discriminant analysis (OPLS-DA) was then utilized to visualize the metabolic difference. As shown in Figure 2B, a good discrimination between the two groups was achieved by OPLS-DA scores plot. The predictive ability of the model was measured by internal validation (R^2^Y= 0.791, Q^2^ = 0.601, CV-ANOVA *p*-value < 0.01), suggesting that the model possessed a satisfactory fit with good predictive power, and the metabolite differences between the groups within the model were highly significant. A random permutation test (200 times) of the corresponding OPLS-DA model was performed to further evaluate the robustness of this model, as exhibited by the steep R^2^ and Q^2^ regression lines and small difference between R^2^ and Q^2^ (R^2^Y = 0.791, Q^2^ = 0.601), indicating that it is a good model suitable for data analysis (Figure 2C). To further assess the prediction ability of the model to unknown samples, 80% of samples (“training set”, healthy controls = 26, CRC = 54) were randomly selected to construct OPLS-DA model, which was then used to predict the remaining 20% of samples (“testing set”, healthy controls = 6, CRC = 14). As can be seen in Figure 2D, healthy controls of the testing set were correctly located in the region of healthy controls from the training set, and the same results were obtained in the testing set of CRC samples.

**Figure 3:**
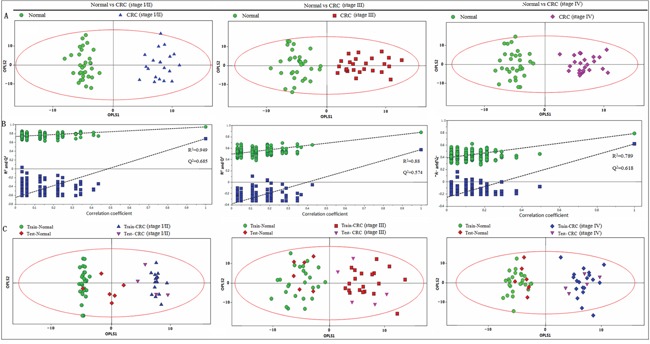
PR analysis of 1H-NMR fecal spectra between different stages of CRC and healthy control **A.** OPLS-DA scatter plot based on healthy controls and each stage of CRC, green dots represent healthy control (n = 32); blue triangles represent stage I/II (n = 20); red boxes represent stage III (n = 25) and purple diamonds represent stage IV (n = 23). **B.** statistical validation of the corresponding PLS-DA model by permutation analysis (200 times). **C.** scores plot of OPLS-DA prediction model. 80% of samples were applied to construct the model, and then used it to predict the remaining 20% of samples (“testing set”, healthy controls = 6; stage I/II = 4; stage III = 5; stage IV = 5). Red diamonds represent healthy controls and purple inverted triangles represent CRC.

OPLS-DA was applied to distinguish the differences of fecal profiles between healthy controls and each stage of CRC. The scores plot indicated that each stages (I/II, III and IV) of CRC could be clearly separated from healthy controls (Figure 3A). Model parameters of permutation analysis for different stages were as follows: stage I/II: R^2^Y = 0.949, Q^2^ = 0.685; stage III: R^2^Y = 0.880, Q^2^ = 0.574 and stage IV: R^2^Y= 0.789, Q^2^ = 0.618, which indicated the good fit obtained by the model (Figure 3B). The training and testing set evaluations further validated the predictive power of the model. The testing set samples were correctly classified as either CRC group or healthy controls (Figure 3C).

### Metabolites contributing to the CRC fingerprint for early detection

**Figure 4:**
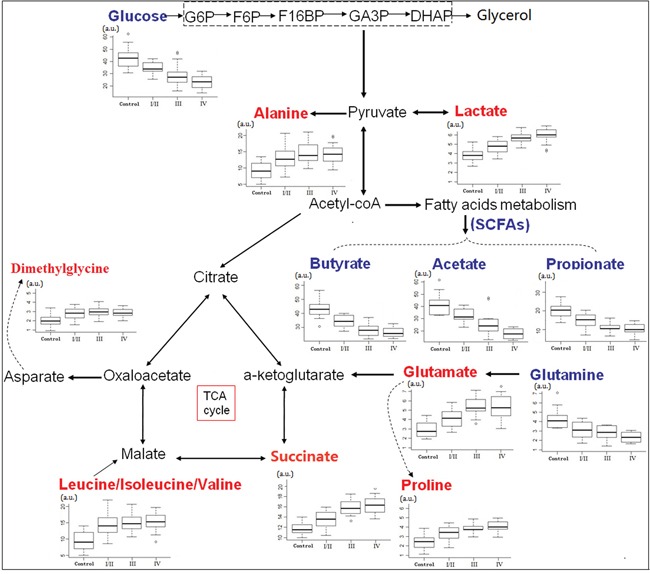
Metabolic network of the significantly changed metabolites involved in glycolysis, TCA cycle and amino acid metabolism Box-and-whisker plots of metabolites that showed progressive changes over different CRC stages relative to healthy controls. Horizontal line in the middle portion of the box, median; bottom and top boundaries of boxes, lower and upper quartile; whiskers, 5th and 95th percentiles; open circles, outliers. Red text = increased with respect to control, blue text = decreased.

Fecal metabolites that met the following conditions were considered as potential biomarker candidates for the earlier detection of CRC: the levels of metabolites with variable importance in the projection (VIP) > 1 and the presence of a significant difference (*p*<0.05) between metabolite levels of the stage I/II CRC patients and healthy controls according to the Mann-Whitney U test. Lower fecal levels (*p* < 0.05) of SCFAs (acetate, propionate and butyrate), glucose and glutamine and higher metabolite levels (*p* < 0.05) of proline, succinate, isoleucine, leucine, valine, alanine, glutamate, dimethylglycine and lactate were present in the feces of stage I/II CRC patients, as compared to the healthy controls (Table 1). Leucine, isoleucine and valine overlap at 0.94-9.99 ppm and were described as “leucine/isoleucine/valine” in this manuscript. Furthermore, the altered fecal metabolites from the different pathological stages of CRC were obtained, and the metabolomics of feces at stage I/II differed markedly from those at later stages (p < 0.05) (Figure 4).

**Table 1:** Resonance intensity ratios, standard deviation, *p* values, sensitivity, specificity, accuracy, AUROC and cut-off value of the metabolites whose levels differed significantly between the stage I/II CRC patients and healthy controls

Metabolite (peak position)	Relative intensity (a.u.)			Sensitivity	Specificity	Accuracy	AUROC (95% CI)	Cut-off value
	control	CRC	*p* value					
Butyrate (0.90ppm)	23 ± 6	14±6↓	0.001	69.2	84.6	76.9	0.843 (0.692-0.995)	20
(1.56ppm)	20 ± 78	12±5↓	0.002	72.1	92.3	80.7	0.828 (0.666-0.991)	18
Acetate (1.92ppm)	45 ±11	24±6↓	<0.001	94.7	92.3	93.6	0.985 (0.949-1.021)	33.5
Propionate (1.06ppm)	26 ± 7	19±6↓	0.005	53.8	100	72.5	0.787 (0.612-0.962)	25
Leucine/Isoleucine/Valine (0.95-0.99ppm)	10 ± 2	13±2↑	0.001	100	46.5	73.1	0.84 (0.692-0.989)	10.5
Alanine (1.48ppm)	9.0± 2	12±2↑	0.001	76.9	99.2	84.6	0.864 (0.712-1.016)	11.5
Dimethylglycine (2.8ppm)	3.2±1	4.3±1↑	0.01	69.2	79.6	73.1	0.775 (0.594-0.956)	3.5
proline (3.34ppm)	2.3± 1	4.1±1↑	<0.001	74.3	92.3	83.3	0.845 (0.676-0.993)	3.35
succinate (2.41ppm)	12 ± 1	15±2↑	<0.001	91.2	93.5	92.3	0.935 (0.820-1.050)	13.7
Glucose (3.42-3.9ppm)	37±7	26±7↓	0.001	69.2	100	84.6	0.888 (0.763-1.012)	34.5
Lactate (1.33ppm)	4.8± 1	6.9±2↑	0.003	84.6	76.9	80.7	0.855 (0.699-1.011)	5.9
Glutamate (2.35ppm)	3.2 ± 2	4.9±1↑	<0.001	61.5	84.6	80.7	0.849 (0.693-1.006)	4.6
Glutamine (2.45ppm)	4.5± 1	2.7±1↓	<0.001	92.3	76.9	84.6	0.908 (0.795-1.022)	3.25

## DISCUSSION

^1^H NMR spectroscopy-based metabolomics of human feces offers two important opportunities: first, the chance to investigate CRC-associated metabolic alterations that may serve as biomarkers, and second, the fecal profile obtained may provide us with an invaluable insight into the pathogenesis of the disease. There have only been a few reports of fecal metabolic changes associated with CRC to date; previous ^1^H NMR-based metabolomic studies already suggested fecal metabolic alterations between CRC patients and healthy controls [26, 27]. However, none has described early changes to the fecal metabolic profile. Our study was designed to investigate different patterns between stages of CRC patients compared to healthy controls, and to identify patients with early stage (stage I/II). Cross validation, model permutations, training and testing evaluations were performed to validate the predictive accuracy of the multivariate ^1^H NMR model. Our findings revealed that the fecal metabolic profiles of healthy controls can be well discriminated from those of even early stage (stage I/II) CRC patients (Figure 3). In addition, glucose, lactate, SCFAs, glutamate and succinate at stage I/II differed markedly from those at stages III and IV (Figure 4), which provided the molecular information associated with the staging of CRC. Our findings indicated that the difference in fecal NMR spectral profiles between diseased and non-diseased patients faithfully depicts the pathophysiological changes and metabolic disturbances observed at the different phases of the disease progression, highlighting the benefits of NMR-based fecal metabolomics as a potential noninvasive strategy to identify biomarkers for CRC earlier diagnosis.

The ^1^H NMR spectral data of fecal extracts contain rich diagnostic information, however conventional analysis fails to utilize this valuable information to a full extent. Pattern recognition technologies provide the potential of analyzing NMR data in a robust, non-subjective and reliable manner. Preliminary unsupervised PCA revealed a partial separation between the CRC patients and healthy controls (Figure 2A). The lack of complete separation between the two groups was not unexpected, as the large inter-individual variability including diet, lifestyle, and gender differences might dilute observable changes from the disease-related ‘metabotype’. To circumvent the systematic variation unrelated to pathological status and optimize class separation, a supervised OPLS-DA was employed [34], which facilitated interpretation by separately modeling predictive and orthogonal (non-predictive) variance. Here, the OPLS-DA demonstrated satisfactory modeling and predictive capabilities for the dataset, revealing a distinct separation between diseased and non-diseased samples (Figure 2B–2D, Figure 3), suggesting that the presence or absence of CRC is an important factor driving the variability in stool metabolites. The sensitivity and specificity of the biomarker candidates were assessed using an ROC analysis, to provide summaries of the predictive performance of the potential biomarkers for earlier detection of CRC (Table 1). As indicated, among the potential biomarkers, acetate (1.92 ppm) and succinate (2.41 ppm) displayed relatively high sensitivity, specificity and accuracy (with a value of larger than 90%, respectively) to distinguish early stage CRC patients from healthy controls; a possible reason for this might be due to the fact that they are singlet peaks with relatively high signal intensity, incurred less overlap with other metabolites, and therefore were more accurately quantified by integration.

Although the onset mechanisms involved in CRC are yet to be fully elucidated, and it is not currently possible to make a conclusive link between carcinogenesis in the colon to a single function, fecal metabolic derangements derived from both neoplasia and gut microbes may culminate in a distinct metabolic phenotype that characterizes the pathology of CRC [35]. SCFAs are microbial-derived metabolites, which are readily absorbed and used as an energy source by colonocytes [36, 37]. SCFAs reduce epithelial in flammation and trigger cancer cell apoptosis via p21 activity [12, 38], providing an important defensive capacity against colorectal carcinogenesis. The observed depletion of SCFAs in feces might propose a disruption of intestinal microbiota and host tissues, associated with colorectal tumourigenesis. Compared to healthy controls, an increase in amino acids, such as leucine, isoleucine, valine, alanine and dimethylglycine, were present in the feces of CRC patients (Figure 4); this could be accounted for by the malabsorption of nutrients due to epithelium inflammation and injury resulting from a particular bowel disease in CRC patients [35]. Increased lactate with an equivalent decrease of glucose levels observed in CRC stool samples might be a result of increased glycolysis to maintain tumor promotion. The increased glucose consumption of tumor tissue leads to a decrease in fecal glucose concentration in parallel to an increase in lactate, consistent with increased energy metabolism due to tumor cell activation, and this observation is supported by an elevation in succinate and glutamate indicative of an increase in TCA cycle activity. A similar pattern of nutrient consumption and by product release was also observed with glutamine consumption, closely mirrored by glutamate release, making it available for gluconeogenesis or for subsequent conversion to other amino acids. Glutaminase activity, a series of biochemical reactions by which glutamine is lysed to glutamate, is another main pillar for energy production in proliferating cells, including colonocytes [29, 39]. Higher levels of proline in the feces of CRC patients might be shed from the tumor, reflecting degradation of intestinal mucins covering the colonic epithelium [40].

Little information is available regarding the main causes of CRC occurrence for the early stages of the relevant time course. Avoidance of early risk accumulation may occur 20 or more years before the projected onset of symptomatic disease [41]. Studies have showed that changes of molecular and biochemical metabolism occur prior to the morphologic alterations [42]. Hence, identifying the characteristic metabolic phenotype prior to the colorectal epithelial malignant transformation would enable the early recognition and intervention of the disease, as well as preventing or delaying the development of CRC. Our study observed distinct NMR-based fecal metabolic signatures which were capable of discriminating early stage (stage I/II) CRC patients from healthy controls. However, whether the fecal metabolomics dysfunction in the early stage of CRC could reflect the characteristic metabolic differences in tumor biology still remains elusive and warrants further study. Moreover, Marchesi et al [43] has observed reduced butyrate, acetate, methylamine and trimethylamine, accompanied by elevated quantities of amino acids in the feces of inflammatory bowel disease (IBD) patients. On the basis of the current findings, it will be of considerable interest to determine the metabolic alterations between normal and cancerous tissues of the same individuals, which could provide evidence linking to the fecal metabolic phenotype. Further experiments will be required to delineate the contributions of IBD and colonic polyps (precancerosis of CRC) on the fecal metabolic perturbations.

A challenge of fecal NMR metabolomics is to extract useful information from a complex sample that contains various species of bacteria, the end products of the digestive processes, and epithelial cells shed from the colorectal mucosa. Food intake could potentially change fecal metabolomic. It is impractical to standardize the diets of every individual. Since we are pursuing common differences when all cancer-harboring patients are compared to all normal controls, on average, the bias of diet should be minimal. Our metabolic findings observed reduced SCFAs accompanied by altered amino acids in the feces of CRC patients, which bear some resemblance to prior reports by Bezabeh et al. [26] and Monleon et al. [27], suggesting that the altered fecal metabolic profiles resulting from bowel disease should be much more significant compared to the variations due to dietary, environmental and genetic factors. In addition, we did not observe tissue specific biomarkers at the downstream fecal level. The discordant set of tissue and fecal markers implied that the unique ‘metabotype’ differentiating exfoliated tumor cells from normal colonocytes was possibly diluted in the feces by the abundant shedding of normal colonocytes. In other words, processes beyond the direct shedding of tumor cells possibly defined the fecal metabotype of CRC patients more extensively.

In summary, in this study, ^1^H NMR spectroscopy coupled with OPLS-DA convincingly demonstrated that the fecal metabolic profiles in CRC patients at an early stage were distinct from those of healthy controls. The altered fecal metabolites potentially revealed disruption of the normal bacterial ecology, malabsorption of nutrients, increased glycolysis, and glutaminolysis, which may be correlated with the initiation and progression of CRC, and may extend our understanding of colonic molecular pathogenesis underlying disease processes.

## MATERIALS AND METHODS

### Ethics statement

The study was approved by the Ethics Committee of Shantou University Medical College. The human fecal samples were used in accordance with the guidelines of Shantou University Affiliated Hospital. Written informed consent was obtained from each subject prior to participating in the study.

### Clinical population

A total of 100 fecal samples collected from May 2014 to December 2014 at the second affiliated hospital of Shantou University Medical College were used in this study, consisting of 32 healthy controls (15M, 17F, age 57±23) and 68 from CRC patients (36M, 32F, age 56±21). The CRC patients were diagnosed by microscopy, biopsy, or surgical resection, and the disease stage was determined according to the American Joint Committee on Cancer (AJCC) [44] staging system for colorectal tumors: stage I/II, 20 patients; stage III, 25 patients; stage IV, 23 patients. None of the cancer patients had any complicating diseases. Healthy controls exhibited no abnormalities from blood tests, endoscopic examination, diagnostic imaging, and/or medical interview. Exclusion criteria for all participants included use of antibiotics, NSAIDS, statins, or probiotics within two months of study participation. Additional exclusion for CRC patients included chemotherapy or radiation treatments prior to surgery. The demographic and clinical characteristics of the patients and healthy controls studied by ^1^H NMR are summarized in Table 2.

**Table 2:** Summary of clinical and demographic characteristics for CRC patients and healthy controls

	CRC patients	healthy controls	*x*^2^ value	*p* value
Number	68	32		
Gender			0.32	0.571
Male	36 (52.9)	15 (46.8)		
Female	32 (47.1)	17 (53.2)		
Age at diagnosis, years			0.928	0.819
<40	8(11.2)	3 (9.4)		
40-49	22 (32.3)	12 (37.5)		
50-59	25 (36.8)	13 (40.6)		
≥60	13 (19.1)	4 (12.5)		
weight(kg)			0.499	0.919
<50	6(8.8)	4 (12.5)		
50-59	29 (42.7)	13 (40.6)		
60-69	26 (38.2)	11 (34.4)		
≥70	7 (10.3)	4 (12.5)		
Cancer stage				
stage-I/II	20			
stage-III	25			
stage-IV	23			

### Fecal sample preparation

All fecal samples were collected immediately after being voided and subsequently stored at −80 ^°^C until further extraction. Frozen stool samples were thawed at room temperature and shaken before use. A total of 3 mL of PBS/D_2_O buffer (0.1 M, *p*H=7.4) was added to 1 g of each feces sample, and the mixture was homogenized by vortexing for 60 s and then centrifuged at 10,000 rpm for 10 minutes. Subsequently, a volume of 500 μL of the supernatant was transferred into an Eppendorf vial, to which 50 μL of a stock solution of sodium (3-trimethylsilyl)-2, 2, 3, 3-tetradeuteriopropionate (TSP) /D_2_O was added, making the final concentration of 4 mM in TSP. The TSP is used as a chemical shift reference (0.0 ppm) and for spectra alignment. Finally, the resulting mixture was centrifuged at 10 000 rpm for 10 minutes, and 450 μL of the supernatant was transferred into a 5 mm high-resolution NMR tube (Wilmad, Buena, NJ, USA) for ^1^H NMR spectroscopic analysis.

### ^1^H NMR spectroscopy

All samples were analyzed on a Bruker AVII 400 MHz NMR spectrometer (Bruker Biospin, Germany) operating at a ^1^H frequency of 400.13 MHz. Magnetic field homogeneity was optimized by gradient or manual shimming prior to acquisition. The temperature was maintained at 298 K and lock performed on the D_2_O signal.^1^H NMR spectra were obtained from a one-dimensional NOESY (nuclear overhauser enhancement spectroscopy) pulse sequence [RD-90°-*t_1_*-90°-*t_m_*-90°-ACQ], with the following acquisition parameters: Recycle Delay, RD = 1.5 s; *t_1_*_=_ 3 μs; mixing time, *t_m_*=100 ms; 90° pulse width=7.3 μs; number of scans, NS=64; number of points, TD=16380; spectral width, SW=5000 Hz; acquisition time, AQ=1.47 s. Water suppression was achieved by irradiation of the water peak during RD and *t_m_*.

### ^1^H NMR spectral data processing

All free induction decays (FIDs) from 1D ^1^H NMR were multiplied by a 0.3 Hz exponential line broadening prior to Fourier Transformation. ^1^H NMR spectra were then corrected for phase and baseline distortion and calibrated to TSP at 0.0 ppm using TOPSPIN (V 2.0, Bruker Biospin). To reduce the complexity of the NMR data, the spectral range from 9.5 to 0.5 ppm was segmented into buckets with the equal width of 0.002 ppm using the AMIX package (V 3.8.3, Bruker Biospin, Germany). The region of 5.2–4.4 ppm was discarded to eliminate imperfect water suppression. Each bucket was internally normalized to the total integral of the spectrum prior to pattern recognition analysis to eliminate the dilution or bulk mass differences among samples due to the different sample weight.

### Pattern recognition (PR) analysis and cross validation

To establish a global overview of the differential characteristics of the CRC patients with respect to healthy controls, multivariate data analysis was applied to the ^1^H NMR data. The normalized NMR spectral data sets were unit variance scaled, and then analyzed using the SIMCA-P+ program (version 14.1, Umetrics AB; Umeå, Sweden). First, a preliminary PCA model was carried out on the mean-centered normalized ^1^H NMR spectra to detect the general trends and outliers. Data were visualized by means of PC scores plots where each point represents an individual sample. Following PCA, OPLS-DA was applied to the analysis of ^1^H NMR spectral data scaled to unit variance. OPLS-DA was also applied to distinguish the differences of fecal profiles between healthy controls and each stage of CRC. The model quality was evaluated with the R^2^Y and Q^2^ values, reflecting the explained fraction of variance and the model predictability. R2Y scores range between 0 and 1 and Q2 scores range between negative and 1, where an R^2^Y score of 1 demonstrates that the model explains 100% of variance, and a Q^2^ score closer to 1 indicates higher reliability of the prediction in the cross-validation procedure. Validation of the OPLS-DA model was also performed by means of a permutation test (200 times). The R^2^Y in the permutated plot describes how well the data fit with the derived model, whereas Q^2^ describes the predictive ability of the derived model (Q^2^>0.5 considered as ‘good’ and Q^2^>0.9 considered as ‘excellent’). The VIP values of all peaks from OPLS-DA models were taken as a coefficient for peak selection, and those variables with VIP>1 were considered as potential biomarker candidates for group discrimination [45].

### Statistical analysis

The relative concentrations of those metabolites with VIP > 1 were calculated by integrating the signals in the spectra. Statistical significance was assessed using the Mann-Whitney U test and a *p* < 0.05 was considered statistically significant. To further evaluate the diagnostic power of the potential biomarkers whose levels differed significantly between the stage I/II CRC patients and healthy controls, receiver operating characteristic (ROC) analysis in SPSS 16.0 was carried out, and the optimal cut-off value, the area under the ROC curve (AUROC), specificity, sensitivity, and accuracy of the metabolites were calculated, where AUROC > 0.8 indicated excellent diagnostic ability.
